# Enhanced Dynamic Laterality Based on Functional Subnetworks in Patients with Bipolar Disorder

**DOI:** 10.3390/brainsci13121646

**Published:** 2023-11-27

**Authors:** Dandan Li, Jiangping Hao, Jianchao Hao, Xiaohong Cui, Yan Niu, Jie Xiang, Bin Wang

**Affiliations:** College of Computer Science and Technology, Taiyuan University of Technology, Jinzhong 030600, China; haojiangping0480@link.tyut.edu.cn (J.H.);

**Keywords:** bipolar disorder, fMRI, dynamic laterality index, laterality time series

## Abstract

An ocean of studies have pointed to abnormal brain laterality changes in patients with bipolar disorder (BD). Determining the altered brain lateralization will help us to explore the pathogenesis of BD. Our study will fill the gap in the study of the dynamic changes of brain laterality in BD patients and thus provide new insights into BD research. In this work, we used fMRI data from 48 BD patients and 48 normal controls (NC). We constructed the dynamic laterality time series by extracting the dynamic laterality index (*DLI*) at each sliding window. We then used k-means clustering to partition the laterality states and the Arenas–Fernandez–Gomez (AFG) community detection algorithm to determine the number of states. We characterized subjects’ laterality characteristics using the mean laterality index (MLI) and laterality fluctuation (LF). Compared with NC, in all windows and state 1, BD patients showed higher MLI in the attention network (AN) of the right hemisphere, and AN in the left hemisphere showed more frequent laterality fluctuations. AN in the left hemisphere of BD patients showed higher MLI in all windows and state 3 compared to NC. In addition, in the AN of the right hemisphere in state 1, higher MLI in BD patients was significantly associated with patient symptoms. Our study provides new insights into the understanding of BD neuropathology in terms of brain dynamic laterality.

## 1. Introduction

Bipolar disorder (BD) is a chronic mental disorder caused by disturbances in the functioning of the brain, characterized by alternating episodes of depression and hypomania [1,2]. BD severely affects more than 1% of the world’s population and is characterized by lifelong episodes [3]. BD can cause severe mental distress and is also life-threatening [4,5]. In recent years, more and more studies have focused on the lesions of brain diseases, and BD patients have received much attention [6,7]. The mining of brain properties in BD patients can help provide beneficial information for disease prevention and treatment.

Functional laterality of the brain, which is the functional asymmetry of the hemispheres, plays an important role in the identification of mental disorders [8]. As an effective neuroimaging index, fMRI has been widely used to study the laterality of brain function in recent years [7,9,10]. Especially in the exploration of the laterality of brain function in BD patients, some studies have shown abnormal changes in the laterality of brain function in BD patients. For example, asymmetric functional connectivity changes have been reported in BD patients [11,12,13]; some of these altered brain regions play important roles in specific subnetworks, and it also indicates how specific functions in the corresponding subnetworks may be affected as a result. Research has also reported that BD patients have laterality changes in brain activation signals at the level of subnetworks and pointed out the correlation between such changes and different emotional symptoms [14,15,16]. According to the above, we can understand the phenomenon of laterality changes in the brain of patients with BD, and these studies provide crucial evidence for the neurodevelopmental mechanism of patients with BD. However, these studies have studied the brain from a static perspective, ignoring the dynamic interaction process of the brain over time.

It is well known that the state of the brain is a dynamic pattern that changes over time [17]. The capture of dynamic changes can help us obtain more effective potential information [18]. Therefore, capturing the dynamic changes of the brain from fMRI is a key initiative in BD research [19,20]. The dynamic patterns of interactions in the brain of BD patients could be effectively distinguished from those of healthy people [21,22], and the corresponding lesions could be manifested in specific subnetworks [23,24]. In terms of brain state transition patterns, BD patients have also been reported to exhibit abnormalities related to subnetworks and increased instability of cognitive processes [25,26]. In addition, some studies have pointed out that BD patients have abnormal neurobiological features of changes in spontaneous brain activity signals [27]. These studies have shown that dynamic features play a crucial role in the expression of BD patients, and the capture of dynamic information is conducive to us exploring BD disease pathology effectively. Based on these studies, we hypothesized that the laterality of the human brain changes over time, and it is very meaningful to explore it for BD research.

This paper aims to consider the brain properties from the perspective of temporal dynamics, and thus to explore how the brain laterality of BD patients has changed. At the same time, functional subnetworks of the brain play an important role in BD research [28]. Therefore, the focus of this paper is to analyze whether the brain subnetwork of BD patients shows corresponding lesions by mining the changes in brain dynamic laterality. Given the above large number of studies, the brain dynamic and brain laterality manifestations of BD contribute to the diagnosis of the disease. Therefore, further exploration of the dynamic changes of laterality will help to provide more accurate clinical diagnosis guidance. We used fMRI data from 48 BD patients and 48 age- and sex-matched normal controls (NC) to construct laterality time series, respectively. We assessed dynamic laterality in BD patients using two indices, namely the mean laterality index (MLI) and laterality fluctuation (LF), to determine the differences between the groups. We explored not only across all windows but also in different time clusters. The number of states was determined by the k-means clustering algorithm and Arenas–Fernandez–Gomez (AFG) community detection algorithm. We also investigated the correlation of abnormal dynamic laterality changes with the clinical features of BD patients. The study of brain dynamic laterality in BD patients may provide a new perspective on the study of brain injury in BD patients.

## 2. Materials and Methods

### 2.1. Subjects

All data included in this work were acquired from the University of California Los Angeles (UCLA) Consortium for Neuropsychiatric Phenomics (CNP) LA5c Study project, and the study was approved by the UCLA Institutional Review Board; data are available through the OpenfMRI database (accession number: ds000030, https://legacy.openfmri.org/dataset/ds000030/) [29]. Finally, we obtained 48 BD patients and 48 matched NCs, which is shown in Table 1. Patient symptoms were assessed using the Brief Psychiatric Rating Scale (BPRS), the Scale for Assessment of Negative Symptoms (SANS), and the Young Mania Rating Scale (YMRS) [30,31,32].

### 2.2. Data Acquisition and Data Processing

Functional MRI data for all participants were collected on a 3T Siemens Trio scanner, using a T2*-weighted echoplanar imaging (EPI) sequence. The relevant parameters are as follows: repetition time (TR) = 2000 ms, echo time (TE) = 30 ms, flip angle = 90°, slice thickness = 4 mm, slice number = 34 and 152 time points. Resting-state fMRI data were preprocessed using the DPARSF toolbox [33]. The first 10 volumes were discarded, to ensure that the subject was acclimated to the environment and the scanner was calibrated [34]. The subsequent steps included slice timing, realign, head motion correction, and normalization to the Montreal Neurological Institute (MNI) space (resampled into 3 × 3 × 3 mm^3^ voxels); a Gaussian kernel of 4 mm full width at half height (FWHM) was used for smoothing. Data were linearly detrended and regressed out the nuisance covariates, including white matter, cerebrospinal fluid signal, and Friston-24 parameters of head motion. Finally, the data were temporally filtered (0.01 ≤ f ≤ 0.1 Hz), which can effectively reflect neuronal fluctuations [35].

### 2.3. Laterality Time Series Construction and Analysis

We used the dynamic laterality index (*DLI*) and adopted a sliding window approach to capture the dynamics of laterality to construct laterality time series [36]. The formula for calculating the *DLI* for window *t*:

(1) $${DLI}^{t}=r\left({ROI}_{i}^{t},{GS}_{L}^{t}\right)-r\left({ROI}_{i}^{t},{GS}_{R}^{t}\right)$$

The *i*th region of interest is Pearson correlated with the left hemisphere global signal or the right hemisphere global signal, respectively. The fisher-z transformation was then performed, and the difference between the above two was calculated.

**Figure 1:**
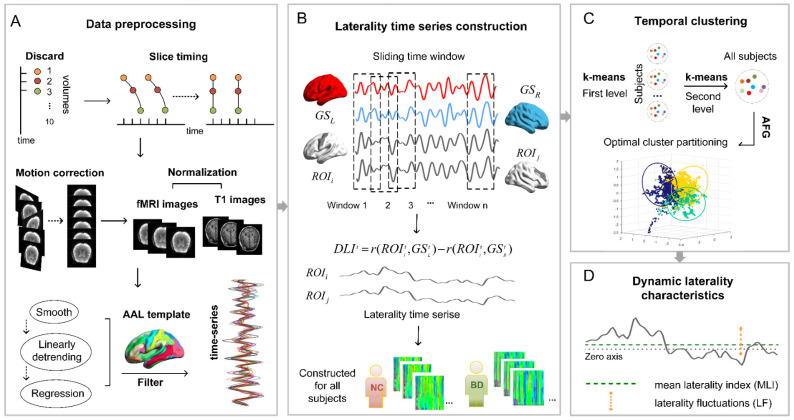
Schematic diagram of the data preprocessing and dynamic laterality analysis process. (**A**) The data preprocessing process included removing the first 10 volumes, slice timing, head movement correction, normalization, smoothing, linear detrending, regression of excess covariates, etc. Finally, the fMRI time series of the corresponding *ROI* were extracted by matching them to the AAL template. (**B**) The *DLI* of each *ROI* was calculated within each window by the sliding window method. Laterality time series were constructed for all *ROI* of all subjects by window sliding. (**C**) The laterality time series of all subjects were clustered using a combination of k-means clustering algorithm and AFG community detection algorithm. The AFG community detection algorithm can help us to determine the optimal number of clusters, namely the k value. (**D**) The mean laterality index and laterality fluctuation were calculated to extract the dynamic laterality characteristics of the subjects. *GS_L_*, global signal of the left hemisphere; *GS_R_*, global signal of the right hemisphere; *ROI*, region of interest. *DLI*, dynamic laterality index; MLI, mean laterality index; LF, laterality fluctuation. AFG, Arenas-Fernandez-Gomez. NC, normal controls; BD, bipolar disorder.

The entire rs-fMRI time series was segmented into several overlapping sub-time series by the sliding window method. The *DLI* of different brain regions in each window was evaluated. The comparison of window sizes indicates that the minimum window length should not be less than the inverse of the minimum frequency of the time series [37]. Therefore, we chose a window size of 50 TR and a window step size of 1 TR, to capture the rapid dynamic changes of the brain more securely. Thus, the 142 time points of each subject were divided into 93 windows (Figure 1B).

We calculated two measures of dynamic laterality. The MLI represents the mean laterality index, which is obtained by averaging over all Windows. LF represents laterality fluctuation, which is the standard deviation across all Windows (Figure 1D). The cortical volume of the template brain was parcellated into 90 regions of the AAL atlas [38] (Figure 1A), and each region in the network is assigned to 5 (Division within hemispheres) subnetworks based on the network division of the network proposed by Yong H et al. [39].

### 2.4. Temporal State Extraction and Analysis

To identify the state of lateral performance across time in the brain, we determined the final mean pattern category as the centroid through a three-stage temporal clustering method. We first clustered the laterality time series for everyone using the k-means clustering algorithm, and then cluster the centroids obtained above. The final number of clusters was determined using an Arenas–Fernandez–Gomez (AFG) community detection algorithm with multi-resolution screening capabilities. In the k-means algorithm, the distance metric we chose was the cosine distance metric. Because laterality time series based on different brain regions and time windows will contain far more features than the sample size, we chose the cosine similarity measure which is more suitable for measuring higher dimensional data [40,41,42]. The AFG community detection algorithm was chosen, with a resolution parameter from 0.1 to 1.5 and a step size of 0.1 [43]. In this paper, according to the existing research on the general state number determination and parameter selection of temporal clustering, the parameter range was selected to ensure that the reliable physiological significance of the division could be obtained [17,36]. In this process, we look for the most persistent (i.e., the most stable) division across multiple scales, resulting in the optimal number of clusters. Finally, cluster centroids obtained during the appeal process were applied separately to all participants, resulting in all brain laterality state partitions (Figure 1C).

### 2.5. Statistical Analysis

To avoid the influence of irrelevant variables on the experiment, we performed an analysis of group differences in demographic and clinical data. Demographic and clinical data were analyzed using SPSS version 25.0 (IBM Corp., Armonk, NY, USA). Group differences in demographic information, including age, education, and handscore were analyzed with two-tailed *t*-tests. We used a χ^2^ test to analyze the gender data. For dynamic laterality parameters, a two-sample two-tailed *t*-test was used to analyze differences between BD patients and NC. A threshold of *p* < 0.05 was considered to indicate significance for between-group differences. Especially for subnetwork or brain region attributes, the false discovery rate (FDR) correction was performed at this threshold (*p* < 0.05).

For those dynamic laterality parameters that showed statistically significant group differences, we used Spearman correlation to analyze the relationship between dynamic laterality characteristics and BD symptom severity. When the uncorrected *p* value was 0.05, a significant relationship was considered. This was because these correlations are exploratory in nature.

## 3. Results

### 3.1. Demographic and Clinical Data

Demographic data are shown in Table 1. There were no significant differences in age (*p* = 0.436), gender (*p* = 0.837), education level (*p* = 0.350), or handscore (*p* = 0.760) between the BD group and the NC group.

### 3.2. Dynamic Laterality Analysis under All Windows

#### 3.2.1. MLI and LF Analysis Based on Subnetworks

**Figure 2:**
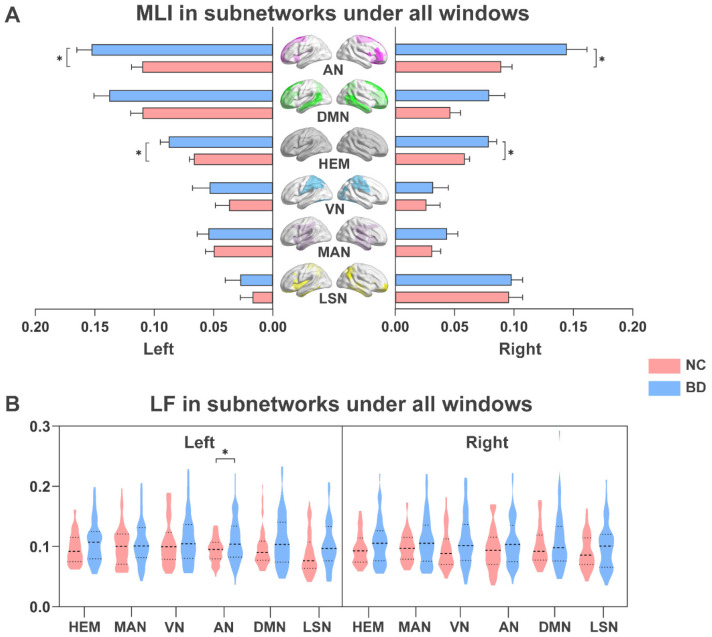
Group differences in dynamic laterality characteristics of subnetworks under all windows. (**A**) MLI for NC and BD. The bar chart and error bar represent the average values and standard deviations of the MLI in each group. (**B**) LF for NC and BD. NC, normal controls; BD, bipolar disorder. MLI, mean laterality index; LF, laterality fluctuation. HEM, Hemisphere; MAN, Somatosensory/Motor and Auditory Network; VN, Visual Network; AN, Attention Network; DMN, Default Mode Network; LSN, Limbic/Paralimbic and Subcortical Network. * represents significant differences between different groups; the *p* value indicates the significance. Two-sample *t*-test, FDR-corrected, * *p* < 0.05.

As shown in Figure 2, we analyzed changes in dynamic laterality in BD patients under all windows. The subnetworks that showed significant differences between LF and MLI features were found. Compared with NC, BD patients showed a higher MLI in bilateral hemisphere networks (*p* < 0.05; Figure 2A). BD patients also had higher MLI in the AN of the left and right hemispheres (*p* < 0.05; Figure 2A). At the same time, as shown in Figure 2B, the attention network (AN) in the left hemisphere of BD patients also exhibited more frequent LF (*p* < 0.05).

#### 3.2.2. Node-Based LF Analysis

**Figure 3:**
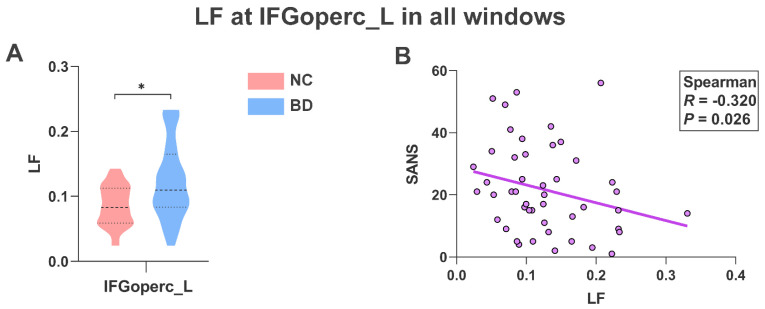
LF analysis of lFGoperc_L. (**A**) LF of NC and BD at the IFGoperc_L node. * represents significant differences between different groups; two-sample *t*-test; FDR-corrected; * *p* < 0.05. (**B**) Spearman correlation between LF and SANS in BD patients; the *R* value indicates the correlation coefficient and the *p* value indicates the significance. NC, normal controls; BD, bipolar disorder. LF, laterality fluctuation. IFGoperc_L, operculum part of the left inferior frontal gyrus. SANS, Scale for Assessment of Negative Symptoms.

As shown in Figure 3A, BD patients showed higher LF (*p* < 0.05) at the operculum part of the left inferior frontal gyrus (IFGoperc_L) than in NC. At the same time, we found that the LF of BD patients in the IFGoperc_L region was significantly negatively correlated with SANS (*R* = −0.320, *p* = 0.026; Figure 3B).

### 3.3. Dynamic Laterality Analysis under Temporal Clustering

#### 3.3.1. The States Obtained by Clustering

**Figure 4:**
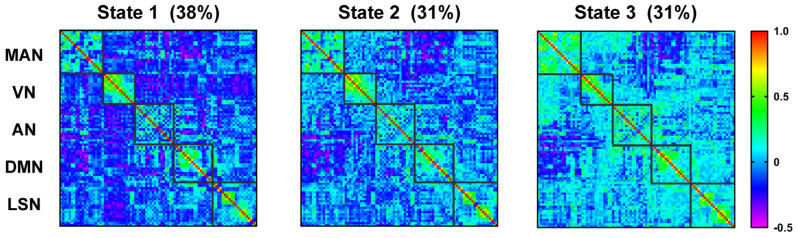
The laterality correlation matrix of states 1–3. At the top, the proportions of different states are shown. On the right, the color bars show the extent of positive and negative correlations for laterality. MAN, Somatosensory/Motor and Auditory Network; VN, Visual Network; AN, Attention Network; DMN, Default Mode Network; LSN, Limbic/Paralimbic and Subcortical Network.

We further explored the temporal organization of laterality dynamics by clustering the whole brain laterality state of time windows. Three laterality states were identified, each showing distinct laterality patterns (Figure 4). Among them, the more negative the laterality correlation is, the more opposite the tendency in which different regions interact with the two hemispheres. Depending on the main manifestations of the different states, are referred to as the contralateral interaction state, the transition state, and the ipsilateral interaction state, respectively.

#### 3.3.2. Analysis of Group Differences Based on Status

**Figure 5:**
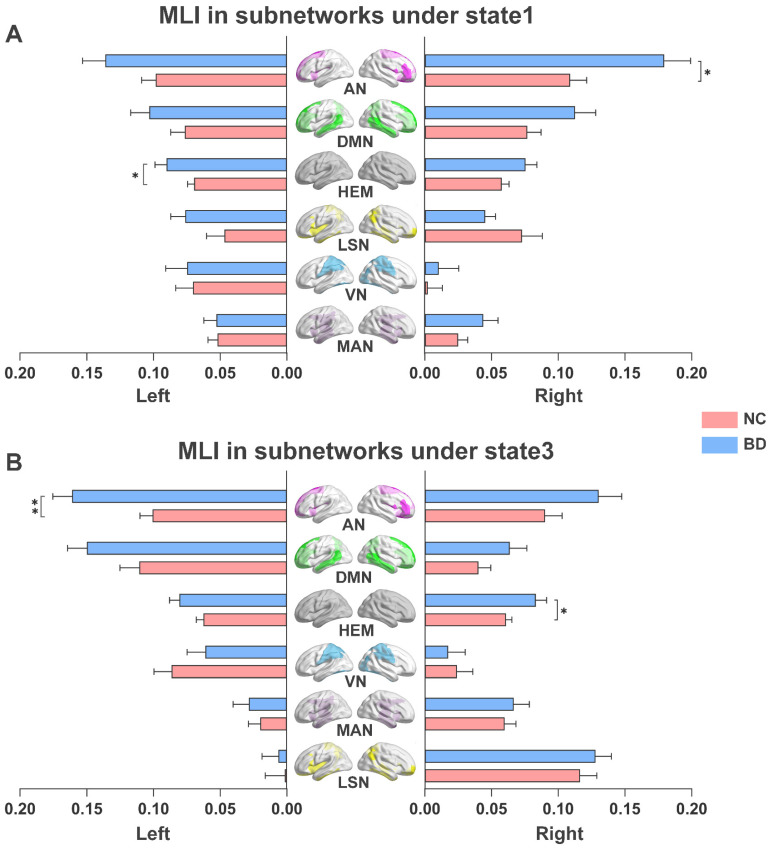
Group differences in MLI of subnetworks under different states. The bar chart and error bar represent the average values and standard deviations of the MLI in each group. (**A**) MLI for NC and BD in state 1. (**B**) MLI for NC and BD in state 3. NC, normal controls; BD, bipolar disorder. MLI, mean laterality index. HEM, Hemisphere; MAN, Somatosensory/Motor and Auditory Network; VN, Visual Network; AN, Attention Network; DMN, Default Mode Network; LSN, Limbic/Paralimbic and Subcortical Network. * Represents significant differences between different groups; the *p* value indicates the significance. Two-sample *t*-test; FDR corrected; * *p* < 0.05; ** *p* < 0.01.

**Figure 6:**
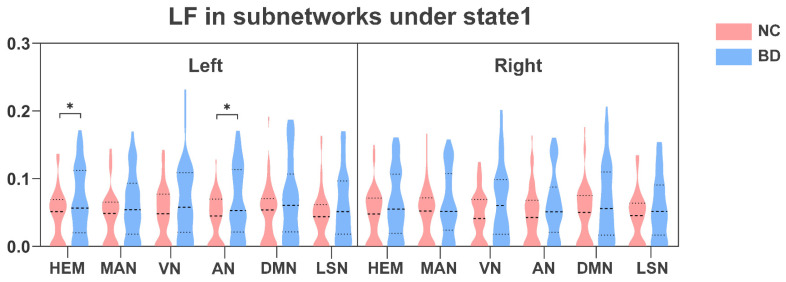
Group differences in LF of subnetworks under state 1. LF, laterality fluctuation. HEM, Hemisphere; MAN, Somatosensory/Motor and Auditory Network; VN, Visual Network; AN, Attention Network; DMN, Default Mode Network; LSN, Limbic/Paralimbic and Subcortical Network. * Represents significant differences between different groups; the *p* value indicates the significance. Two-sample *t*-test; FDR corrected; * *p* < 0.05.

**Figure 7:**
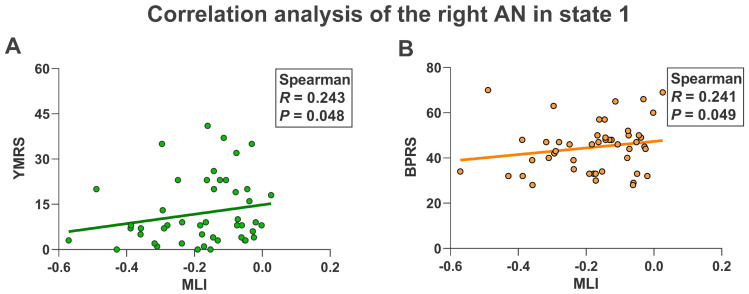
Spearman correlation coefficient between MLI of right AN in state 1 and clinical features of BD patients. (**A**) Related to YMRS. (**B**) Related to BPRS. MLI, mean laterality index. AN, attention network. YMRS, Young Mania Rating Scale. BPRS, Brief Psychiatric Rating Scale. The *R* value indicates the correlation coefficient and the *p* value indicates the significance.

As shown in Figure 5A, compared with NC, the left hemisphere (*p* < 0.05) and the AN of the right hemisphere (*p* < 0.05) in BD patients showed higher MLI in state 1. Furthermore, the right hemisphere (*p* < 0.05) and the AN of the left hemisphere (*p* < 0.05) in BD patients also showed higher MLI in state 3 (Figure 5B). In addition, as shown in Figure 6, NC in state 1 also showed lower LF in the left hemisphere (*p* < 0.05) and the AN in the left hemisphere (*p* < 0.05) than BD patients. Finally, in state 1 we found that the MLI of AN in the right hemisphere in BD patients was significantly positively correlated with YMRS (*R* = 0.243, *p* = 0.048) and BPRS (*R* = 0.241, *p* = 0.049) (Figure 7).

## 4. Discussion

We explored time-resolved alterations in functional lateralization, which was not only across all time windows but also across time clusters. We mainly reported dynamic laterality changes characteristic in the presence of subnetworks in BD patients, and some correlated with symptom scales. We found that these differences are mainly concentrated in the AN of both hemispheres, indicating the potential role of AN in the genetic risk and phenotypic expression of BD.

### 4.1. The Increased MLI of the AN in the Left Hemisphere Is Caused by Weakened Inter-Hemispheric Connectivity

Across all windows, we found that the MLI of AN in the left hemisphere was enhanced in BD patients. According to the calculation principle of MLI, we speculate that this was caused by the decreased inter-hemispheric connectivity of the AN in the left hemisphere in BD patients. Previous research [44,45,46] has reported that in patients with BD, the AN in the left hemisphere showed weaker functional connectivity between hemispheres. Several studies [47,48] have suggested that the AN in the left hemisphere plays an important role in motor attention and language-related functions, while studies [14,49] also point to impaired motor attention and other functions in BD patients. 

From the perspective of changes in brain state, this paper further found that AN in the left hemisphere of BD patients in state 3 exhibited higher MLI. This result further supports the results of all windows above, indicating that MLI abnormalities in the left hemisphere AN are simultaneously caused mainly by state 3. State 3 represents ipsilateral interaction, and according to the principle of ipsilateral interaction, the brain is likely to be engaged in the processing of simple tasks [36,50]. These simple tasks involve the processing of some motor attention functions and language functions [51,52,53], which also supports the possible impairment of corresponding functions in BD patients mentioned in the appeal. In summary, the above evidence suggests that the increased MLI of AN in the left hemisphere in BD patients is caused by weakened inter-hemispheric connectivity and occurs mainly in specific ipsilateral interaction states. The results suggest that the impairment of AN in the left hemisphere may provide a new direction for the diagnosis of BD.

### 4.2. The Increased MLI of AN in the Right Hemisphere Is Caused by Enhanced Intra-Hemispheric Connectivity

Across all windows, BD patients were found to exhibit greater MLI in the AN of the right hemisphere. Also, according to the calculation principle of MLI, we suspect that the AN in the right hemisphere is more likely to interact with its hemisphere, due to its stronger connections within the hemisphere. There is evidence of enhanced intra-hemispheric functional connectivity of AN in the right hemisphere in BD patients [12,54,55,56], accompanied by similar findings in structural connectivity [57,58]. The AN in the right hemisphere tends to play an important role in spatial attention and executive control, which may reflect a compensatory mechanism for related functions [59,60,61]. We found that the triggers of MLI changes were different in the AN of the bilateral hemispheres, and some studies [62,63] have pointed out the asymmetry of the AN itself. 

From the perspective of brain state changes, we further found that AN in the right hemisphere of BD patients exhibited higher MLI in state 1. This result further supports the above results under all windows, indicating that the MLI abnormalities of AN in the right hemisphere are mainly caused in state 1. State 1 represents the contralateral interaction, and according to the principle of contralateral interaction, the brain is in the process of more complex tasks currently [36,64]. In the contralateral interaction state, some studies [65] have also pointed out that BD patients have the performance of task-related integration enhancement. These complex tasks involve processing related to spatial attention and executive control [66,67]. Given the above discussion, we conclude that the MLI abnormality of the AN in the right hemisphere of BD patients is caused by its increased intra-hemispheric connectivity, and mainly occurs in the state of contralateral interaction. This finding further complements the diagnostic value of AN impairment in the right hemisphere in BD patients.

### 4.3. The Increased LF of AN in the Left Hemisphere Was Caused by Enhanced Connectivity Variability

Across all windows, AN in the left hemisphere in BD patients showed LF more frequently than NC. And according to the calculation principle of LF, we speculate that is caused by more frequent changes in connectivity between the AN in the left hemisphere and the rest of the brain. Previous work [68,69,70] has shown that in patients with BD, AN in the left hemisphere showed increased variability in dynamic functional connections with the motor cortex and the default mode network. Many studies [45,71,72] have pointed out that the IFGoperc region in this network tends to be altered in response to brain injury, as well as in BD patients. Similarly, we also found abnormally frequent LF changes in left IFGoperc. We speculate that the strong laterality fluctuations in BD patients play a compensatory role, to compensate for the lack of information exchange between the AN of the left hemisphere and other regions [73]. This result ties well with previous studies [74,75,76], wherein BD patients have compensatory mechanisms in the AN, which can enhance the processing ability of attention information. The left IFGoperc is an important region in the processing of phonological-related working memory mechanisms [77,78]. The above phenomena reflect the attention-related functional compensation that may arise from brain damage in BD patients, especially in language-related working memory. 

In addition, in state 1, namely the contralateral interaction state, BD patients also showed frequent changes in LF in the AN of the left hemisphere. According to the previous discussion, the brain in the state of contralateral interaction is in the process of highly busy complex tasks [36,79]. At this time, the AN in the left hemisphere of BD patients also showed more frequent information exchange. Obviously, in the state of contralateral interaction, to make up for its shortcomings and meet the needs of many information exchanges, it shows abnormal compensation behavior [80,81,82,83]. In summary, we conclude that the higher LF in the AN of BD patients in the left hemisphere is a compensatory mechanism that occurs mainly in the contralateral interaction state. This conclusion may provide consideration for the clinical treatment of BD from the perspective of compensation mechanisms.

### 4.4. Relationship with Clinical Features

This study investigated the relationship between abnormal dynamic laterality features and symptom severity in BD patients. In state 1, namely the contralateral interaction state, the MLI performance of AN in the right hemisphere of BD patients was positively correlated with YMRS and BPRS. YMRS and BPRS reflect the severity of manic and psychotic symptoms, respectively; higher YMRS and BPRS lead to more serious attacks. According to the existing studies, we believe that this may lead to reduced flexibility of attention switching in BD patients during clinical manifestations, leading to more severe seizures [6,84,85,86]. This evidence suggests that higher MLI in the right AN during bilateral interaction states could reflect more severe disease onset in BD patients. At the same time in all windows, the MLI of the IFGoperc region in the left hemisphere showed a negative correlation trend with the SANS. We suggest that this negative correlation may be due to enhanced connectivity variability in the left IFGoperc. That is, a lower SANS score indicates a higher frequency of connection communication. This is clearly a compensatory behavior for impaired attention; the abnormal manifestations in this region also reflect the severity of the disease in BD patients [71]. Our correlation results may provide evidence for alterations related to attentional function in BD patients.

## 5. Limitations

This study also has some limitations, mainly due to the small sample size of the data. Although we obtained relatively stable and reliable results, the universality of the sample still needs to be explored and improved by recruiting more sample sizes in future surveys.

## 6. Conclusions

In this study, we explored the abnormal brain dynamic laterality in BD patients using fMRI. We found that the AN in the left hemisphere of BD patients exhibited higher MLI across all Windows, resulting from its reduced inter-hemispheric connectivity, and predominantly in state 3. The AN in the right hemisphere of BD patients showed higher MLI across all Windows, which was caused by its enhanced connectivity in the right hemisphere, and this change mainly occurred in state 1. The AN in the left hemisphere in BD patients showed more frequent LF across all Windows, which we indicated was caused by frequent dynamic connection changes with other regions, and mainly occurred in state 1. Finally, we also found that in state 1, the abnormal MLI of the AN in the right hemisphere was positively correlated with clinical features of BD patients. Overall, the dynamic laterality of AN in both hemispheres was significantly altered in BD patients, which was closely related to attention-related functional abnormalities in BD patients. Our study provides novel fMRI markers of brain lesions in BD patients and is expected to provide strong support for clinical decision making.

## Figures and Tables

**Table 1:** Demographic and clinical characteristics (mean ± standard deviation [SD]).

Subjects	NC (*n* = 48)	BD (*n* = 48)	*p*
Age (years)	33.77 ± 9.0	35.23 ± 9.0	0.436 ^a^
Gender (M/F)	26/22	27/21	0.837 ^b^
Education level(years)	14.94 ± 1.5	14.60 ± 1.9	0.350 ^a^
Handscore ^c^	0.93 ± 0.1	0.93 ± 0.1	0.760 ^a^
BPRS ^d^	N/A	44.73 ± 10.9	N/A
SANS ^d^	N/A	21.63 ± 14.1	N/A
YMRS ^d^	N/A	12.02 ± 11.0	N/A

Unless otherwise indicated, all data are represented as mean  ±  standard deviation. ^a^ The *p* value was obtained using a two-sample two-tailed *t*-test. ^b^ The *p* value was obtained using a χ^2^ test. ^c^ The Handscore described the handedness of subjects. It was obtained using a formula (Right − Left)/(Right ± Left). ^d^ The BPRS, SANS, and YMRS scores were used to assess the symptom severity of patients with BD.

## Data Availability

Data are contained within the article.

## References

[1] Claeys E.H.I., Mantingh T., Morrens M., Yalin N., Stokes P.R.A. (2022). Resting-state fMRI in depressive and (hypo)manic mood states in bipolar disorders: A systematic review. Prog. Neuro-Psychopharmacol. Biol. Psychiatry.

[2] Ping L.L., Zhou C., Sun S., Wang W.Q., Zheng Q., You Z.Y. (2022). Alterations in resting-state whole-brain functional connectivity pattern similarity in bipolar disorder patients. Brain Behav..

[3] Grande I., Berk M., Birmaher B., Vieta E. (2016). Bipolar disorder. Lancet.

[4] Poletti S., Bollettini I., Mazza E., Locatelli C., Radaelli D., Vai B., Smeraldi E., Colombo C., Benedetti F. (2015). Cognitive performances associate with measures of white matter integrity in bipolar disorder. J. Affect. Disord..

[5] Vieta E., Berk M., Schulze T.G., Carvalho A.F., Suppes T., Calabrese J.R., Gao K., Miskowiak K.W., Grande I. (2018). Bipolar disorders. Nat. Rev. Dis. Primers.

[6] Brady R.O., Tandon N., Masters G.A., Margolis A., Cohen B.M., Keshavan M., Ongur D. (2017). Differential brain network activity across mood states in bipolar disorder. J. Affect. Disord..

[7] Yin W., Li L., Wu F.-X. (2022). Deep learning for brain disorder diagnosis based on fMRI images. Neurocomputing.

[8] Xie W., Peng C.-K., Huang C.-C., Lin C.-P., Tsai S.-J., Yang A.C. (2018). Functional brain lateralization in schizophrenia based on the variability of resting-state fMRI signal. Prog. Neuro-Psychopharmacol. Biol. Psychiatry.

[9] Luckett P.H., Maccotta L., Lee J.J., Park K.Y., Dosenbach N.U.F., Ances B.M., Hogan R.E., Shimony J.S., Leuthardt E.C. (2022). Deep learning resting state functional magnetic resonance imaging lateralization of temporal lobe epilepsy. Epilepsia.

[10] White L.K., Makhoul W., Teferi M., Sheline Y.I., Balderston N.L. (2023). The role of dlPFC laterality in the expression and regulation of anxiety. Neuropharmacology.

[11] Vizueta N., Rudie J.D., Townsend J.D., Torrisi S., Moody T.D., Bookheimer S.Y., Altshuler L.L. (2012). Regional fMRI hypoactivation and altered functional connectivity during emotion processing in nonmedicated depressed patients with bipolar II disorder. Am. J. Psychiatry.

[12] Torrisi S., Moody T.D., Vizueta N., Thomason M.E., Monti M.M., Townsend J.D., Bookheimer S.Y., Altshuler L.L. (2013). Differences in resting corticolimbic functional connectivity in bipolar I euthymia. Bipolar Disord..

[13] de Almeida J.R.C., Versace A., Mechelli A., Hassel S., Quevedo K., Kupfer D.J., Phillips M.L. (2009). Abnormal amygdala-prefrontal effective connectivity to happy faces differentiates bipolar from major depression. Biol. Psychiatry.

[14] Romeo Z., Marino M., Angrilli A., Semenzato I., Favaro A., Magnolfi G., Padovan G.B., Mantini D., Spironelli C. (2022). Altered language network lateralization in euthymic bipolar patients: A pilot study. Transl. Psychiatry.

[15] Caligiuri M.P., Brown G.G., Meloy M.J., Eyler L.T., Kindermann S.S., Eberson S., Frank L.R., Lohr J.B. (2004). A functional magnetic resonance imaging study of cortical asymmetry in bipolar disorder. Bipolar Disord..

[16] Huang K., Kang Y., Wu Z., Wang Y., Cai S., Huang L. (2021). Asymmetrical alterations of grey matter among psychiatric disorders: A systematic analysis by voxel-based activation likelihood estimation. Prog. Neuro-Psychopharmacol. Biol. Psychiatry.

[17] Casorso J., Kong X., Chi W., Van De Ville D., Yeo B.T., Liégeois R. (2019). Dynamic mode decomposition of resting-state and task fMRI. NeuroImage.

[18] Handwerker D.A., Roopchansingh V., Gonzalez-Castillo J., Bandettini P.A. (2012). Periodic changes in fMRI connectivity. Neuroimage.

[19] Espinoza F.A., Anderson N.E., Vergara V.M., Harenski C.L., Decety J., Rachakonda S., Damaraju E., Koenigs M., Kosson D.S., Harenski K. (2019). Resting-state fMRI dynamic functional network connectivity and associations with psychopathy traits. Neuroimage Clin..

[20] Li W.C., Wang C.Y., Lan X.F., Fu L., Zhang F., Ye Y.X., Liu H.Y., Wu K., Zhou Y.L., Ning Y.P. (2022). Variability and concordance among indices of brain activity in major depressive disorder with suicidal ideation: A temporal dynamics resting-state fMRI analysis. J. Affect. Disord..

[21] Rashid B., Damaraju E., Pearlson G.D., Calhoun V.D. (2014). Dynamic connectivity states estimated from resting fMRI Identify differences among Schizophrenia, bipolar disorder, and healthy control subjects. Front. Hum. Neurosci..

[22] Wang H., Zhu R.X., Tian S., Shao J.N., Dai Z.P., Xue L., Sun Y.R., Chen Z.L., Yao Z.J., Lu Q. (2022). Classification of bipolar disorders using the multilayer modularity in dynamic minimum spanning tree from resting state fMRI. Cogn. Neurodyn..

[23] Rey G., Bolton T.A.W., Gaviria J., Piguet C., Preti M.G., Favre S., Aubry J.M., Van De Ville D., Vuilleumier P. (2021). Dynamics of amygdala connectivity in bipolar disorders: A longitudinal study across mood states. Neuropsychopharmacology.

[24] Wang H., Zhu R., Tian S., Zhang S., Dai Z., Shao J., Xue L., Yao Z., Lu Q. (2022). Dynamic connectivity alterations in anterior cingulate cortex associated with suicide attempts in bipolar disorders with a current major depressive episode. J. Psychiatr. Res..

[25] Wang J., Wang Y., Huang H., Jia Y., Zheng S., Zhong S., Huang L., Huang R. (2019). Abnormal intrinsic brain functional network dynamics in unmedicated depressed bipolar II disorder. J. Affect. Disord..

[26] Du M., Zhang L., Li L., Ji E., Han X., Huang G., Liang Z., Shi L., Yang H., Zhang Z. (2021). Abnormal transitions of dynamic functional connectivity states in bipolar disorder: A whole-brain resting-state fMRI study. J. Affect. Disord..

[27] Zhang N., Niu Y., Sun J., An W., Li D., Wei J., Yan T., Xiang J., Wang B. (2021). Altered Complexity of Spontaneous Brain Activity in Schizophrenia and Bipolar Disorder Patients. J. Magn. Reson. Imaging.

[28] Nabulsi L., McPhilemy G., Kilmartin L., Whittaker J.R., Martyn F.M., Hallahan B., McDonald C., Murphy K., Cannon D.M. (2020). Frontolimbic, frontoparietal, and default mode involvement in functional dysconnectivity in psychotic bipolar disorder. Biol. Psychiatry Cogn. Neurosci. Neuroimaging.

[29] Poldrack R.A., Barch D.M., Mitchell J.P., Wager T.D., Wagner A.D., Devlin J.T., Cumba C., Koyejo O., Milham M.P. (2013). Toward open sharing of task-based fMRI data: The OpenfMRI project. Front. Neuroinform..

[30] Andreasen N.C. (1990). Methods for assessing positive and negative symptoms. Mod. Probl. Pharmacopsychiatry.

[31] Overall J.E., Gorham D.R. (1962). The Brief Psychiatric Rating Scale. Psychol. Rep..

[32] Young R.C., Biggs J.T., Ziegler V.E., Meyer D.A. (1978). A rating scale for mania: Reliability, validity and sensitivity. Br. J. Psychiatry.

[33] Yan C.-G., Wang X.-D., Zuo X.-N., Zang Y.-F. (2016). DPABI: Data processing & analysis for (resting-state) brain imaging. Neuroinformatics.

[34] Zang Y., Jiang T., Lu Y., He Y., Tian L. (2004). Regional homogeneity approach to fMRI data analysis. Neuroimage.

[35] Conio B., Magioncalda P., Martino M., Tumati S., Capobianco L., Escelsior A., Adavastro G., Russo D., Amore M., Inglese M. (2019). Opposing patterns of neuronal variability in the sensorimotor network mediate cyclothymic and depressive temperaments. Hum. Brain Mapp..

[36] Wu X., Kong X., Vatansever D., Liu Z., Zhang K., Sahakian B.J., Robbins T.W., Feng J., Thompson P., Zhang J. (2022). Dynamic changes in brain lateralization correlate with human cognitive performance. PLoS Biol..

[37] Leonardi N., Van De Ville D. (2015). On spurious and real fluctuations of dynamic functional connectivity during rest. Neuroimage.

[38] Tzourio-Mazoyer N., Landeau B., Papathanassiou D., Crivello F., Etard O., Delcroix N., Mazoyer B., Joliot M. (2002). Automated anatomical labeling of activations in SPM using a macroscopic anatomical parcellation of the MNI MRI single-subject brain. Neuroimage.

[39] He Y., Wang J., Wang L., Chen Z.J., Yan C., Yang H., Tang H., Zhu C., Gong Q., Zang Y. (2009). Uncovering intrinsic modular organization of spontaneous brain activity in humans. PLoS ONE.

[40] Dhillon I.S., Modha D.S. (2001). Concept decompositions for large sparse text data using clustering. Mach. Learn..

[41] Vohryzek J., Deco G., Cessac B., Kringelbach M.L., Cabral J. (2020). Ghost attractors in spontaneous brain activity: Recurrent excursions into functionally-relevant BOLD phase-locking states. Front. Syst. Neurosci..

[42] Wang Y., Li Z., Wang Y., Wang X., Zheng J., Chen H. (2015). A Novel Approach for Stable Selection of Informative Redundant Features from High Dimensional Feature Spaces. arXiv.

[43] Arenas A., Fernandez A., Gomez S. (2008). Analysis of the structure of complex networks at different resolution levels. New J. Phys..

[44] Roberts G., Lord A., Frankland A., Wright A., Lau P., Levy F., Lenroot R.K., Mitchell P.B., Breakspear M. (2017). Functional dysconnection of the inferior frontal gyrus in young people with bipolar disorder or at genetic high risk. Biol. Psychiatry.

[45] Lv D., Lin W., Xue Z., Pu W., Yang Q., Huang X., Zhou L., Yang L., Liu Z. (2016). Decreased functional connectivity in the language regions in bipolar patients during depressive episodes but not remission. J. Affect. Disord..

[46] Zhao L., Wang Y., Jia Y., Zhong S., Sun Y., Qi Z., Zhang Z., Huang L. (2017). Altered interhemispheric functional connectivity in remitted bipolar disorder: A Resting State fMRI Study. Sci. Rep..

[47] Rushworth M.F., Krams M., Passingham R.E. (2001). The attentional role of the left parietal cortex: The distinct lateralization and localization of motor attention in the human brain. J. Cogn. Neurosci..

[48] Rushworth M.F., Johansen-Berg H., Gobel S.M., Devlin J.T. (2003). The left parietal and premotor cortices: Motor attention and selection. Neuroimage.

[49] Udal A.H., Malt U.F., Lovdahl H., Gjaerum B., Pripp A.H., Groholt B. (2009). Motor function may differentiate attention deficit hyperactivity disorder from early onset bipolar disorder. Behav. Brain Funct..

[50] Esteves M., Lopes S.S., Almeida A., Sousa N., Leite-Almeida H. (2020). Unmasking the relevance of hemispheric asymmetries—Break on through (to the other side). Prog. Neurobiol..

[51] Specogna I., Casagrande F., Lorusso A., Catalan M., Gorian A., Zugna L., Longo R., Zorzon M., Naccarato M., Pizzolato G. (2012). Functional MRI during the execution of a motor task in patients with multiple sclerosis and fatigue. Radiol. Med..

[52] Reynolds J.E., Billington J., Kerrigan S., Williams J., Elliott C., Winsor A.M., Codd L., Bynevelt M., Licari M.K. (2019). Mirror neuron system activation in children with developmental coordination disorder: A replication functional MRI study. Res. Dev. Disabil..

[53] Nettekoven C., Jonas K., Lichtenstein T., Grefkes C., Goldbrunner R., Lucas C.W. (2018). PB7. Comparison of three different fMRI paradigms for language mapping. Clin. Neurophysiol..

[54] Horacek J., Mikolas P., Tintera J., Novak T., Palenicek T., Brunovsky M., Hoschl C., Alda M. (2015). Sad mood induction has an opposite effect on amygdala response to emotional stimuli in euthymic patients with bipolar disorder and healthy controls. J. Psychiatry Neurosci..

[55] Rey G., Piguet C., Benders A., Favre S., Eickhoff S.B., Aubry J.M., Vuilleumier P. (2016). Resting-state functional connectivity of emotion regulation networks in euthymic and non-euthymic bipolar disorder patients. Eur. Psychiatry.

[56] He Z., Sheng W., Lu F., Long Z., Han S., Pang Y., Chen Y., Luo W., Yu Y., Nan X. (2019). Altered resting-state cerebral blood flow and functional connectivity of striatum in bipolar disorder and major depressive disorder. Prog. Neuro-Psychopharmacol. Biol. Psychiatry.

[57] Ji E., Guevara P., Guevara M., Grigis A., Labra N., Sarrazin S., Hamdani N., Bellivier F., Delavest M., Leboyer M. (2019). Increased and Decreased Superficial White Matter Structural Connectivity in Schizophrenia and Bipolar Disorder. Schizophr. Bull..

[58] Heng S., Song A.W., Sim K. (2010). White matter abnormalities in bipolar disorder: Insights from diffusion tensor imaging studies. J. Neural Transm..

[59] Spagna A., Kim T.H., Wu T., Fan J. (2020). Right hemisphere superiority for executive control of attention. Cortex.

[60] DiNuzzo M., Mascali D., Bussu G., Moraschi M., Guidi M., Macaluso E., Mangia S., Giove F. (2022). Hemispheric functional segregation facilitates target detection during sustained visuospatial attention. Hum. Brain Mapp..

[61] Lagopoulos J., Ivanovski B., Malhi G.S. (2007). An event-related functional MRI study of working memory in euthymic bipolar disorder. J. Psychiatry Neurosci..

[62] Spagna A., Martella D., Fuentes L.J., Marotta A., Casagrande M. (2016). Hemispheric modulations of the attentional networks. Brain Cogn..

[63] Bartolomeo P., Seidel Malkinson T. (2019). Hemispheric lateralization of attention processes in the human brain. Curr. Opin. Psychol..

[64] Hartwigsen G., Bengio Y., Bzdok D. (2021). How does hemispheric specialization contribute to human-defining cognition?. Neuron.

[65] Frangou S. (2019). Neuroimaging Markers of Risk, Disease Expression, and Resilience to Bipolar Disorder. Curr. Psychiatry Rep..

[66] Nardo D., Santangelo V., Macaluso E. (2011). Stimulus-driven orienting of visuo-spatial attention in complex dynamic environments. Neuron.

[67] Uddin L.Q. (2021). Cognitive and behavioural flexibility: Neural mechanisms and clinical considerations. Nat. Rev. Neurosci..

[68] Pang Y., Chen H., Wang Y., Long Z., He Z., Zhang H., Liao W., Cui Q., Chen H. (2018). Transdiagnostic and diagnosis-specific dynamic functional connectivity anchored in the right anterior insula in major depressive disorder and bipolar depression. Prog. Neuro-Psychopharmacol. Biol. Psychiatry.

[69] Lu F., Chen Y., Cui Q., Guo Y., Pang Y., Luo W., Yu Y., Chen J., Gao J., Sheng W. (2023). Shared and distinct patterns of dynamic functional connectivity variability of thalamo-cortical circuit in bipolar depression and major depressive disorder. Cereb. Cortex.

[70] Kaiser R.H., Whitfield-Gabrieli S., Dillon D.G., Goer F., Beltzer M., Minkel J., Smoski M., Dichter G., Pizzagalli D.A. (2016). Dynamic Resting-State Functional Connectivity in Major Depression. Neuropsychopharmacology.

[71] Lin X., Zhou R.B., Huang J., Su Y.S., Mao R.Z., Niu Z.A., Cao L., Hu Y.Y., Yang T., Wang X. (2020). Altered resting-state fMRI signals and network topological properties of bipolar depression patients with anxiety symptoms. J. Affect. Disord..

[72] Lan Z., Xu S., Wu Y., Xia L., Hua K., Li M., Liu M., Yin Y., Li C., Huang S. (2021). Alterations of Regional Homogeneity in Preschool Boys With Autism Spectrum Disorders. Front. Neurosci..

[73] Hallam G.P., Thompson H.E., Hymers M., Millman R.E., Rodd J.M., Lambon Ralph M.A., Smallwood J., Jefferies E. (2018). Task-based and resting-state fMRI reveal compensatory network changes following damage to left inferior frontal gyrus. Cortex.

[74] Macoveanu J., Kjaerstad H.L., Vinberg M., Harmer C., Fisher P.M., Knudsen G.M., Kessing L.V., Miskowiak K.W. (2021). Affective episodes in recently diagnosed patients with bipolar disorder associated with altered working memory-related prefrontal cortex activity: A longitudinal fMRI study. J. Affect. Disord..

[75] Fleck D.E., Eliassen J.C., Durling M., Lamy M., Adler C.M., DelBello M.P., Shear P.K., Cerullo M.A., Lee J.H., Strakowski S.M. (2012). Functional MRI of sustained attention in bipolar mania. Mol. Psychiatry.

[76] Sepede G., De Berardis D., Campanella D., Perrucci M.G., Ferretti A., Serroni N., Moschetta F.S., Del Gratta C., Salerno R.M., Ferro F.M. (2012). Impaired sustained attention in euthymic bipolar disorder patients and non-affected relatives: An fMRI study. Bipolar Disord..

[77] Nixon P., Lazarova J., Hodinott-Hill I., Gough P., Passingham R. (2004). The inferior frontal gyrus and phonological processing: An investigation using rTMS. J. Cogn. Neurosci..

[78] Wang J., Yang Y., Zhao X., Zuo Z., Tan L.-H. (2020). Evolutional and developmental anatomical architecture of the left inferior frontal gyrus. NeuroImage.

[79] Cai Q., Van der Haegen L., Brysbaert M. (2013). Complementary hemispheric specialization for language production and visuospatial attention. Proc. Natl. Acad. Sci. USA.

[80] Stern Y., Barnes C.A., Grady C., Jones R.N., Raz N. (2019). Brain reserve, cognitive reserve, compensation, and maintenance: Operationalization, validity, and mechanisms of cognitive resilience. Neurobiol. Aging.

[81] Herbet G., Maheu M., Costi E., Lafargue G., Duffau H. (2016). Mapping neuroplastic potential in brain-damaged patients. Brain.

[82] Syan S.K., Smith M., Frey B.N., Remtulla R., Kapczinski F., Hall G.B.C., Minuzzi L. (2018). Resting-state functional connectivity in individuals with bipolar disorder during clinical remission: A systematic review. J. Psychiatry Neurosci..

[83] Piguet C., Fodoulian L., Aubry J.M., Vuilleumier P., Houenou J. (2015). Bipolar disorder: Functional neuroimaging markers in relatives. Neurosci. Biobehav. Rev..

[84] Bradley A.J., Anderson K.N., Gallagher P., McAllister-Williams R.H. (2020). The association between sleep and cognitive abnormalities in bipolar disorder. Psychol. Med..

[85] Chang Z., Wang X., Wu Y., Lin P., Wang R. (2023). Segregation, integration and balance in resting-state brain functional networks associated with bipolar disorder symptoms. Hum. Brain Mapp..

[86] Zhang L., Li W., Wang L., Bai T., Ji G.J., Wang K., Tian Y. (2020). Altered functional connectivity of right inferior frontal gyrus subregions in bipolar disorder: A resting state fMRI study. J. Affect. Disord..

